# Isolation, characterization, and genomic analysis of a novel lytic bacteriophage with potent antibiofilm activity against multidrug-resistant *Acinetobacter baumannii*

**DOI:** 10.1186/s12866-026-05262-7

**Published:** 2026-07-17

**Authors:** Ethar M. Amin, Ramy K. Aziz, Fathia E. Murad, Marwa T. Elrakaiby, Reham Samir

**Affiliations:** 1 Department of Microbiology and Immunology, Faculty of Pharmacy, Cairo University, Qasr El-Ainy Stree, Cairo, 11562 Egypt; 2 MARC Bioscience Research Laboratories, Giza, Egypt

**Keywords:** Bacteriophage, *Acinetobacter baumannii*, Multidrug-resistant, Genomic analysis, Phage therapy, Antimicrobial resistance

## Abstract

The rapid rise of multidrug-resistant *Acinetobacter baumannii* (MDR *A. baumannii*) poses a serious threat in healthcare settings, as these bacteria can cause infections that are untreatable with conventional antibiotics. This crisis has driven the search for alternative therapies, one of which is bacteriophages. In this study, we isolated a novel bacteriophage, vB_AbaM_EA1 (E7 for short), from wastewater and evaluated its potential activity against clinical MDR *A. baumannii* strains. Phage E7 successfully infects (27.5%) of the 40 clinical MDR *A. baumannii* isolates we tested. Laboratory characterization suggested that E7 is a highly efficient lytic phage, rapidly attaching to bacterial cells and producing 1000 new viral particles.

A key finding was its potent activity against bacterial biofilms, a major source of persistent infections. Not only did Phage E7 effectively prevent the formation of new biofilms, but more importantly disrupted mature ones, which typically confer higher resistance to antibiotics.

Genome sequencing confirmed that Phage E7 is a new member of the *Obolenskvirus* genus and that its genome lacks genes encoding bacterial toxins, antibiotic resistance, or lysogenic factors, indicating a mandatory safety profile for therapeutic use. Furthermore, E7 remained stable across a broad temperature range (-20 to 45 °C) and pH values (5–9) that it might encounter during storage and administration. In conclusion, Phage E7 is a stable, safe, and highly effective candidate for phage therapy against infections by MDR *A. baumannii*. Its ability to target both planktonic bacteria and biofilms makes it a promising candidate for future development into clinical applications.

## Introduction

The World Health Organization has recognized the rapid emergence of multidrug-resistant bacteria (MDRB) as a critical threat to public health [1]. MDRB-related infections are expected to cause nearly 10 million deaths annually by the year 2050 [2]. What adds to the gravity of the matter is that the rate at which new antibiotics are being developed is not sufficient to replace drugs that are becoming less effective over the years [3]. This widening gap between resistance acquisition and antibiotic development has prompted urgent calls for alternative therapeutic strategies that can circumvent conventional resistance mechanisms [4].

*A. baumannii* is a pathogen that causes respiratory and urinary tract infections, secondary meningitis, and sepsis [5]. Its remarkable capacity to survive on dry hospital surfaces for weeks and its ability to acquire resistance determinants through horizontal gene transfer make it exceptionally difficult to control in healthcare settings [4, 6]. Treatment of *A. baumannii* poses a challenge, not only because its strains are resistant to β-lactam antibiotics, but also because many isolates are resistant to other antimicrobial agents, such as carbapenems, aminoglycosides, polymyxins, and tigecycline. Such strains are known as MDR *A. baumannii* [7–11]. Extensively drug-resistant (XDR) and pan-drug-resistant (PDR) phenotypes, against which virtually no antibiotic retains activity, have been increasingly reported across healthcare systems in both high-income and low-income countries, rendering some infections effectively untreatable with currently available antibiotics [12]. A particularly clinically significant feature of *A. baumannii* pathogenicity is its capacity to form robust biofilms on both biotic and abiotic surfaces, including medical devices such as catheters, mechanical ventilators, and endotracheal tubes [13]. Biofilm-embedded cells exhibit tolerance to antibiotics at concentrations 10 to 1,000 times higher than those required to kill planktonic counterparts, largely due to limited antibiotic penetration through the extracellular polymeric substance (EPS) matrix and the presence of metabolically dormant cells [14].

The rate at which *A. baumannii* can develop or acquire resistance to new antibiotics, is higher than that at which new antibiotics can be discovered and developed [15].

Therefore, therapeutic strategies to counter clinical multidrug resistant infections has become limited. This, in turn, calls for developing alternative antibacterial agents and programs [16]. However, the incentive to develop new antibacterial agents is lacking due to the speedy acquisition of resistance by this organism and the increasing cost of production [16, 17].

Phage therapy is an alternative strategy for *A. baumannii* infections, offering a new solution against its dangers [18]. Phages can specifically target only the species and even the strains causing a particular infection [19–21]. This remarkable ability prevents them from affecting human cells and members of the body microbiota [21]. This strain-level specificity, while historically viewed as a limitation, is increasingly recognized as a therapeutic advantage, as it minimizes disruption of the host microbiome and reduces the selective pressure that drives broad-spectrum antibiotic resistance [22]. The development of the phage system is considered to be relatively economic, since phage therapy is highly versatile in terms of phage formulation and applications [3, 23]. Moreover, spontaneous mutations may confer phage–bacteria coevolution, allowing phages to overcome bacterial resistance [24].

Several lytic bacteriophages infecting *A. baumannii* have been isolated and characterized in recent years, demonstrating diverse genomic features and variable host ranges. Despite these advances, several key limitations persist in the literature: many characterized phages infect a narrow range of clinical strains, exhibit limited or untested antibiofilm activity, lack comprehensive genomic safety profiling, or have not been assessed for physicochemical stability under conditions relevant to pharmaceutical storage and administration. There remains, therefore, a critical need for the isolation and rigorous characterization of novel *A. baumannii* phages that combine broad clinical strain coverage with potent antibiofilm activity, favorable genomic safety profiles, and physicochemical stability compatible with therapeutic use.

In terms of application, phages are therapeutic or biocontrol agents that have been in regular use for almost a century in several countries, including Georgia and Russia [25].

Coupled with that, this approach has gained regulatory recognition, with several phage products receiving approval from the US Food and Drug Administration department (US-FDA) [26], and a growing number of documented compassionate-use cases against MDR *A. baumannii* infections have reported clinical success. The landmark case reported by Schooley et al. demonstrated that intravenous administration of a phage cocktail rescued a patient with disseminated MDR *A. baumannii* infection refractory to all antibiotic regimens, marking a turning point in the clinical acceptance of phage therapy in Western medicine [27]. It is worth emphasizing that the use of phages for purposes of therapy necessitates the continuous isolation of phages with novel host specificities to meet the emerging bacterial pathogens that evolve under the never-ending arms race between phages and bacteria.

In the present study, we report the isolation, biological characterization, and comprehensive genomic analysis of a novel lytic bacteriophage, vB_AbaM_EA1 (phage E7), recovered from urban wastewater in Cairo, Egypt. We evaluated its lytic activity against a panel of 40 clinical MDR A. baumannii isolates, determined its kinetic parameters, physicochemical stability, and antibiofilm efficacy against both newly forming and mature biofilms, and performed whole-genome sequencing and phylogenetic analysis to establish its taxonomic classification and confirm its safety profile for potential therapeutic application.

## Materials and methods

### Bacterial isolates identification and growth conditions

Forty strains of *A. baumannii* were isolated from clinical samples, routinely collected in Kasr Al-Aini Hospital in Cairo between 2020 and 2021. The bacterial isolates were initially identified based on culture characteristics and microscopic investigation by Gram staining. To confirm the identification, PCR, targeting the *A. baumannii oxa-*51 gene, was performed. The primers were synthesized by Macrogen, Korea and their sequences are.

FP: 5’- TAATGCTTTGATCGGCCTTG − 3’ and RP: 5’- TGGATTGCACTTCATCTTGG − 3’ [28, 29]. After PCR, the product was separated on 1% (w/v) agarose gel electrophoresis against a 100 bp DNA ladder and the amplicon size was confirmed against standard strain *A. baumannii* ATCC 19,606.

The isolates were cultured in Luria Bertani Broth (LBB) media at 37 °C in a shaking incubator at 180 rpm. For long term storage, the isolates were preserved in LBB media supplemented with 20% glycerol and stored at -80 °C. The stored isolated strains were used as hosts for the isolation of bacteriophages and determination of the host range.

### Antimicrobial susceptibility assay

The antimicrobial susceptibility assay was conducted per Clinical & Laboratory Standards Institute 2020 [30] recommendations to classify the isolates as susceptible, intermediate, or resistant. Ten different antimicrobials were tested, spanning eight distinct antimicrobial classes. The antimicrobial discs used in the study (listed in Table 1) were manufactured by Oxoid. Antimicrobial susceptibility results were subsequently evaluated by the Multiple Antibiotic Resistance Index (MAR) method. The MAR index was calculated as a ratio of the number of resistant antimicrobial agents to the total number of tested agents [31]. This index serves as a valuable tool for monitoring bacterial infections and tracking antimicrobial resistance (AMR).

**Table 1:** List of antimicrobial agents

Antimicrobial agent	Class	Abbreviation	Concentration
Piperacillin-tazobactam	Beta-Lactam Combination	TZP	100/10 µg
Amoxicillin/Clavulanic acid		AMC	30/10 µg
Cefoxitin	2nd Generation Cephalosporin	FOX	30 µg
Cefotaxime	3rd Generation Cephalosporin	CTX	30 µg
Cefepime	4th Generation Cephalosporin	FEP	30 µg
Meropenem	Carbapenem	ME	10 µg
Gentamicin	Aminoglycoside	CN	10 µg
Amikacin		AK	30 µg
Levofloxacin	Fluoroquinolone	LEV	5 µg
Trimethoprim- Sulfamethoxazole	Sulfonamides/Folate Antagonist	SXT	1.25/23.75 µg

### Phage isolation

Wastewater samples were collected from an urban sewage treatment plant in Cairo. Each bacterial isolate was cultured in TSB and incubated overnight at 37 °C under agitation at 180 rpm in an incubator shaker. A mixed culture was assembled from overnight cultures of six isolates (0.5 mL of each) into a 100 mL conical flask with 50 mL of fresh TSB and incubated overnight at 37 °C under agitation at 180 rpm in an incubator shaker. The sewage sample was enriched by adding an equal volume of the mixed culture of *A. baumannii* isolates to a sterile 15 mL falcon centrifuge tube [32–34]. This culture was incubated for 4 h at 37 °C in a static incubator to promote initial phage-host contact and adsorption efficiency during the enrichment phase, centrifuged at 3,467×g for 20 min, and 1 mL of the supernatant was collected and stored at 4 °C until further use. The collected phage lysates from each sewage sample were checked for culturable phages by spotting 10 µL of the lysate twice on Tryptic Soy Agar (TSA, DifcoTM, BD, Franklin Lakes, NJ, USA) plates supplemented with 0.5% (w/v) top layer of soft TSA, containing 100 µL of mid-log phase bacterial inoculant. After overnight incubation at 30 °C, sixty-nine phage plaques were visually identified and excised from bacterial lawns, and then reconstituted in 500 µL sodium magnesium (SM) buffer and stored at 4 °C for further purification and characterization. Of the 69 initially identified phage spots, 40 were excluded from further study based on the following criteria: overlapping or unclear plaque morphology that prevented reliable and reproducible purification, failure to produce stable and consistent plaques after three successive rounds of single-plaque isolation, or insufficient lytic activity against the reference host strain upon repeated testing. The 29 phages that were retained for further characterization were those that reproducibly formed large, clear, well-isolated plaques with consistent lytic activity following purification, characteristics that are indicative of a strictly lytic lifestyle and that are considered standard selection criteria in therapeutic phage development [35, 36].

### Plaque purification, phage propagation and concentration

Initial phage isolates were purified through successive rounds of plaque isolation. Lysates were serially diluted (10-fold) in SM buffer, spotted on dried bacterial lawns, and incubated at 30 °C in aerobic conditions [32–34]. From the top agar layer, single plaques were selected and eluted in SM buffer. Purified stocks were generated after six purification cycles for every isolated bacteriophage [36]. Subsequently, aliquots of purified bacteriophages were used for propagation.

The plate lysate method was used to propagate the purified bacteriophages [37]. Plaques corresponding to the concentrated phage lysates were enumerated by the double agar overlay plaque method [32]. Twenty-nine lytic bacteriophages were retrieved from this step.

### Host range determination

The host range of the purified phages was determined by spotting assay on bacterial hosts relevant to the phage-isolating host species. Their plaques were scored based on clarity into three categories: (1) clear plaque, indicating efficient lytic infection and complete bacterial lysis; (2) turbid or partial plaque, indicating weak or incomplete infection; and (3) no plaque, indicating bacterial resistance or absence of infection [35]. The phage with the broadest host range was picked for further investigation.

### Relative efficiency of plating

Phage E7 was selected for further characterization based on its relatively broad host range among the tested clinical isolates. to evaluate its effectiveness against *A. baumannii* strains, the efficiency of the plating method (EOP) was utilized [38]. EOP was employed as a quantitative complement to the qualitative spot assay, providing a standardized measure of phage productivity across host strains. EOP values reflect the relative replication efficiency of phage E7 on each tested strain. Briefly, Phage E7 was diluted ten-fold, ranging from 10^1^ to 10^8^. A volume of 10 µL from each dilution was applied, in triplicate, onto each susceptible bacterial isolate. After overnight incubation, the number of plaques corresponding to the original phage titer was assessed, and the mean number of spots from the triplicates was calculated. The relative EOP is the ratio of the mean plaque count for the same phage titer on each bacterial host to the highest recorded plaque count [38]. The EOP was categorized into four levels of efficiency, ranging from “high production” to “no production” (Table 2) [39].

**Table 2:** Levels of the relative efficiency of plating

EOP Level	Range of plaque count ratio	
	From	To
High production	≥ 0.5	≥ 1
Medium production	0.1	< 0.5
Low production	> 0.001	< 0.19
No production	0	≤ 0.001

### Morphological identification of phage E7 by electron microscopy

Purified lysate of 10^10^ PFU/mL of the phage was prepared and examined by transmission electron microscopy at the National Research Centre (NRC), Cairo, Egypt core laboratory. 

Micrographs using a High-resolution (HR) transmission electron microscope were generated (JEOL Japan-JEM 2100). The virion dimensions were measured by ImageJ version 1.53n against microscope-generated scale bars. Phage E7 was classified morphologically and taxonomically according to the International Committee on Taxonomy of Viruses 2022 [40].

### *In vitro* bacteriolytic activity (long-term growth inhibition assay)

The bacteriolytic dynamics of Phage E7 was assessed over 24 h across a range of MOIs (10^− 4^ to 10^3^). The reduction in the growth of the bacterial host was used to evaluate the bacteriolytic activity of the phage in a phage-treated culture relative to the untreated (control) bacterial culture. Briefly, for each MOI, a 20 µL mid-log phase bacteria culture achieving a starting concentration of 10⁷ CFU/mL was mixed with 160 µL of fresh LBB in a 96-well plate, yielding a final bacterial concentration of 10⁶ CFU/mL per well. The mixture was inoculated with 20 µL Phage E7 lysate with phage concentrations adjusted to achieve the target MOIs. The plate was incubated at 37 °C, the plate reader was set to agitate for 5 s prior to each reading, and optical density (OD_600_) was recorded every 1 h to monitor lytic progression.

### Phage adsorption and one-step growth curve evaluation

Exponentially grown *A. baumannii* cells were mixed with Phage E7 at an MOI of 1 and incubated at room temperature. Samples (100 µL) were taken at (0, 1, 2, 3, 4, 5, 6, 7, 8, 9, 10, 15, 20, 25 and 30 min), centrifuged (12,900 ×g, 5 min), and then the supernatants containing the unabsorbed phages were titrated [41].

To determine the latent period and burst size of phage E7, a one-step growth curve was performed following previously described methods with minor modifications [36, 42]. The challenged bacteria (10^8^ cfu/mL) were infected with Phage E7 at an MOI of 0.001(corresponding to 10⁵ PFU/mL), a low MOI selected to ensure synchronous single-cycle infection and minimize the probability of multiple phage adsorption to the same host cell. The adsorption process was allowed to occur for 5 min at room temperature. The mixture was centrifuged (12,900 ×g, 1 min), and the pellet was resuspended in 10 mL of fresh LBB and incubated for 1 h with agitation at 30 °C.

The samples were taken at 0, 5, 10, 15, 20, 25, 30, 40, 50 and 60 min. For titer determination, these samples were subsequently diluted and plated by the double-layer agar method. The latent period was defined as the interval between the phages’ adsorption to bacterial cells and their progeny release. The burst size of the phage was calculated as the quotient of the peak phage titer at a plateau phase and the initial infective phages during the latent period.

### Phage stability

The stability of Phage E7 was evaluated under a range of pH and temperature values following previously described methods with minor modifications [36, 43]. A range of temperatures was used to account for both storage and varying thermal conditions, which were (-20 °C, 4 °C, 25 °C, 37 °C, 45 °C, 55 °C and 65 °C). Phage aliquots were prepared and adjusted at 10^10^ PFU/mL as starting titers for each temperature. The phage titers of each aliquot were estimated at specified time intervals for 2 h (15, 30, 60 and 120 min) and tittered after 24 h.

Additionally, Phage E7 stability was assessed throughout a wide pH range of 3 to 11. Phage lysate of titer 10^10^ PFU/mL in SM buffer was prepared with the specific pH values using either 1 M hydrochloric acid (HCl) or 1 M sodium hydroxide (NaOH). After incubating at 4 °C for 1 h and 24 h, the phage titers were measured for each pH value. All phage titers were enumerated by Double Agar Overlay Plaque Assay [32]. Temperature and pH stability experiments were performed in triplicates.

### Bacteriophage potency against bacterial biofilm

The anti-biofilm efficiency of Phage E7 at different MOIs was assessed for both functions: prevention of biofilm formation and disruption of preformed biofilm. The evaluated MOIs were 10^− 7^, 10^− 6^, 10^− 5^ and 10 ^5^. The biofilm formation, staining, and measurement assays were done using the microtiter plate biofilm assay with minor modifications [44, 45].

Exponentially growing bacterial culture (10^5^ CFU/mL in LBB, OD_600_ adjusted) were aliquoted (125 µL/well) into flat-bottomed polystyrene microtiter plates (Greiner Bio-One, Portugal). Each plate included six negative control wells containing untreated bacterial suspension. Phage solutions at specified MOIs were added to test wells immediately (for inhibition studies) or after 48 h of biofilm formation (for eradication studies).

#### Biofilm inhibition assay

Prior to biofilm studies, we conducted a minimum inhibitory concentration (MIC) assessment to distinguish antibiofilm activity from bacteriolytic effects [46]. Bacterial cultures were co-incubated with phages MOIs ranging from 10^− 7^ to 10^− 4^. MOIs with no lytic activity were selected for biofilm studies to ensure observed inhibition reflected antibiofilm activity rather than bacterial killing. Each condition included six replicates. The microtiter plates were incubated at 30 °C and 37 °C for 6 h and 24 h. Biofilm formation was qualified by crystal violet staining and absorbance measurement (595 nm).

#### Biofilm clearance assay

For biofilm clearance studies, mature biofilms were first established by incubating untreated bacterial cultures for 48 h following a previously described protocol [42, 45]. The higher MOI was selected to test whether sufficient phage concentration could penetrate and disrupt the dense extracellular matrix of mature 48-hour biofilms. Phage suspensions at MOIs 10^− 5^ and 10 ^5^ were then added to the pre-formed biofilms and incubated for an additional 6 h and 24 h at 30 °C and 37 °C. To normalize for growth variability, the OD600 of well contents was measured prior to processing. Planktonic cells were removed by washing with tap water, followed by staining with 150 µL of 0.1% (w/v) crystal violet per well. After destaining with 150 µL of 30% acetic acid, biofilm biomass was quantified by measuring OD595 using the Synergy 2 Microplate Reader (BioTek, USA).

### Bacteriophage DNA extraction

Prior to DNA extraction, phage preparations were treated with DNase I (37 °C, 30 min) to eliminate potential bacterial DNA contamination. The treated phages were then concentrated by centrifugation (12,900 ×g, 90 min, 4 °C), and the resulting pellet was processed for nucleic acid extraction. Genomic DNA was isolated from the purified phage preparation using the viral DNA/RNA extraction kit (Thermo Fisher Scientific, USA) following the manufacturer’s instructions. DNA purity and concentration were subsequently determined in a Implen nanophotometer (Munich, Germany).

### Genomic characterization and phylogenetic analysis of phage E7

#### High-throughput sequencing

For genomic library generation, the extracted DNA of Phage E7 was fragmented by the tagmentation-based and PCR-based Illumina DNA Prep kit, with custom IDT 10 bp unique dual indices (UDI) and a target insert size of 320 bp. No further DNA fragmentation or size selection steps were conducted. An Illumina NovaSeq 6000 sequencer was used for sequencing the library in one or more multiplexed shared-flow-cell runs, producing 2 × 151 bp paired-end reads.

Demultiplexing, quality control and adapter trimming were performed with bcl-convert1 (v4.1.5).

#### Quality check and assembly

Raw sequence reads were quality-checked by FastQC (v0.11.9), and adapters were trimmed with Trimmomatic (v0.39). The reads were de novo assembled by Unicycler (v0.5.0), implemented within the BV-BRC portal [34, 35]. The assembly was polished with Pilon (v1.24), and the resulting contig was visualized using Bandage (v0.9.0). Assembly statistics were generated using QUAST (v5.2.0) and SAMtools (v1.17).

#### Genome analysis and annotation

The assembled phage genome was annotated by the RASTtk pipeline [47, 48] with a customized workflow that began with initial annotation via annotate-proteins-phage ⁠ followed by annotate-proteins-kmer-v2 and tRNAscan-SE (v2.0) [49] to identify tRNA genes.

A second-round annotation was performed in the BV-BRC server [50, 51] followed by refinement with DFAST [52]. Pharokka (v1.3) was used to classify genes (e.g., structural, DNA packaging) and identify potential antibiotic resistance genes (ARGs) or virulence factors [53].

Shinefind was used to standardize and expedite the identification of ribosome binding sites (RBS) in the phage’s genome [54].

For functional confirmation, an annotation was performed using NCBI BLASTp against the non-redundant proteins databases, HHPred for remote homology detection [55], InterProScan for domain prediction and UniProt for manual curation. A circular genome map was plotted using Proksee [56].

In the bioinformatics analysis and comparative genomics section, BACPHLIP (Galaxy v1.0) was employed to predict phage lifestyle (lytic/temperate) based upon conserved protein domains [57]. Additionally, PhageLeads predicted therapeutic phage suitability by screening for temperate lifestyle genes, antimicrobial resistance (AMR), and virulence genes [58]. Conserved domains in lytic enzymes (e.g., endolysins) were analyzed using MEME (v5.5.8) [59]. Resistance Gene Identifier (RGI v6.0.5) along with CARD (v4.0.1) [60], and ResFinder (v4.7.2), were used to identify acquired genes and/or finds chromosomal mutations mediating antimicrobial resistance in total or partial DNA sequences. PhageScope [61] and PHASTEST [62] were employed for taxonomic classification and lifestyle prediction (lytic/temperate). Phynteny was used for synteny-based analysis of phage genes, while Phold for sensitive structural annotation of bacteriophage genomes and metagenomes, search against a comprehensive database of over 1 million phage protein structures [63]. The genome maps were plotted using Clinker [64].

For Genome Structure and Proteome Analysis, PhageTerm (Galaxy v1.0.11) determined genome termini and packaging mechanisms [65], while DeepTMHMM (v1.0.11) predicted transmembrane helices in annotated proteins using an algorithm based on a protein language model [66].

The genomic sequence was analyzed by Phage Depolymerase Finder (PhageDPO, Galaxy v0.1.0) by identifying putative depolymerases via the Support Vector Machine (SVM) and the Artificial Neural Network (ANN) models [67].

BLASTn allowed checking the whole-genome sequence of Phage E7 for similarity to deposited sequences in the GenBank-NCBI database. The top hits with the best score, identity (≥ 85%) and probability (e-value ≤ 0) were compared to Phage E7 genome.

#### Taxonomic and phylogenetic analysis

We used the ViPTree server to classify the phage at the family level, comparing it to both closely related strains and other double-stranded DNA prokaryotic viruses at the predicted proteome level, allowing for robust clustering [68]. Phages with the highest 60 ViPTree tBLASTx scores were compared in a rectangular proteomic tree. Moreover, ViPTree was also used for genome-genome comparison of Phage E7 and *Acinetobacter* phages.

Taxon boundaries at the species, genus and family level were estimated with taxMyPhage 3.3.6 [69]. Moreover, for a more specific classification beyond the family level, VIRIDIC, which calculates intergenomic distances among viral genomes, was used to estimate intergenomic similarities between Phage E7 and its closest relatives within the same genus. The nucleotide identity threshold that was applied to differentiate between genera was 70%, and that between species was 95% [70].

Finally, the alignment-free PhageClouds was used to analyze genomic relationships between E7 genome and reference phages genomic sequences in NCBI-GenBank [71]. 

PhageClouds uses dashing to calculates intergenomic distances, based on a threshold of 0.25.

### Statistical analysis

All statistical analyses were performed by GraphPad Prism 10.2 for Mac (GraphPad Software, San Diego, California, USA). Data were represented as means and standard deviations of the mean (± SD). Control and test sets were compared using a t-test/one-way ANOVA at a significance level of *p* < 0.05.

## Results

### Characterization of clinical bacterial isolates

#### Phenotypic and genotypic identification

Gram staining, culture characteristics and biochemical tests indicated that the isolates belonged to the genus *Acinetobacter*. Species-level identification as *A. baumannii* was confirmed by PCR detection of *oxa51*gene, yielding the anticipated amplicon size of 353 bp.

#### Antibiotic resistance profile and mar index of clinical bacterial isolates

**Fig. 1:**
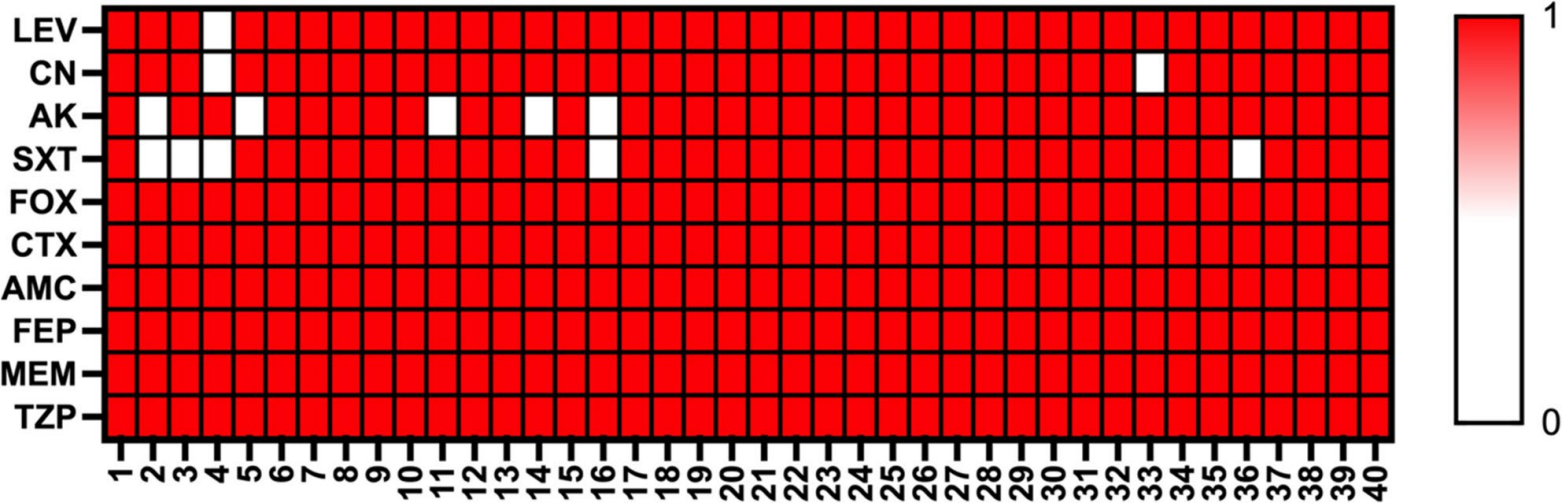
Heatmap of antimicrobial susceptibility testing results for 39 bacterial isolates. The panel represents resistance (red) and susceptibility (white) phenotypes against 10 antimicrobial agents from 8 distinct classes. Agent abbreviations: LEV (Levofloxacin), CN (Gentamicin), AK (Amikacin), SXT (Trimethoprim-Sulfamethoxazole), FOX (Cefoxitin), CTX (Cefotaxime), AMC (Amoxicillin-Clavulanic Acid), FEP (Cefepime), MEM (Meropenem), TZP (Piperacillin-Tazobactam)

Multidrug resistance, defined as resistance to three or more antimicrobial classes, was tested for all 40 clinical isolates. All isolates were indeed resistant to at least three classes (Fig. 1), and their MAR index was above 0.6, confirming their multidrug-resistant phenotype [31]. Remarkably, none of the isolates was susceptible to the antibiotics Cefoxitin, Cefotaxime, Amoxicillin/Clavulanic acid, Cefepime, Meropenem and Piperacillin/Tazobactam. Approximately 13% of the isolates were found to be susceptible to Amikacin and Trimethoprim/Sulfamethoxazole (Fig. 1). The majority of the isolates (> 82%) were resistant to at least two antibiotics from the carbapenem and aminoglycoside classes.

### Phage isolation, host range determination and efficiency of plating

**Fig. 2:**
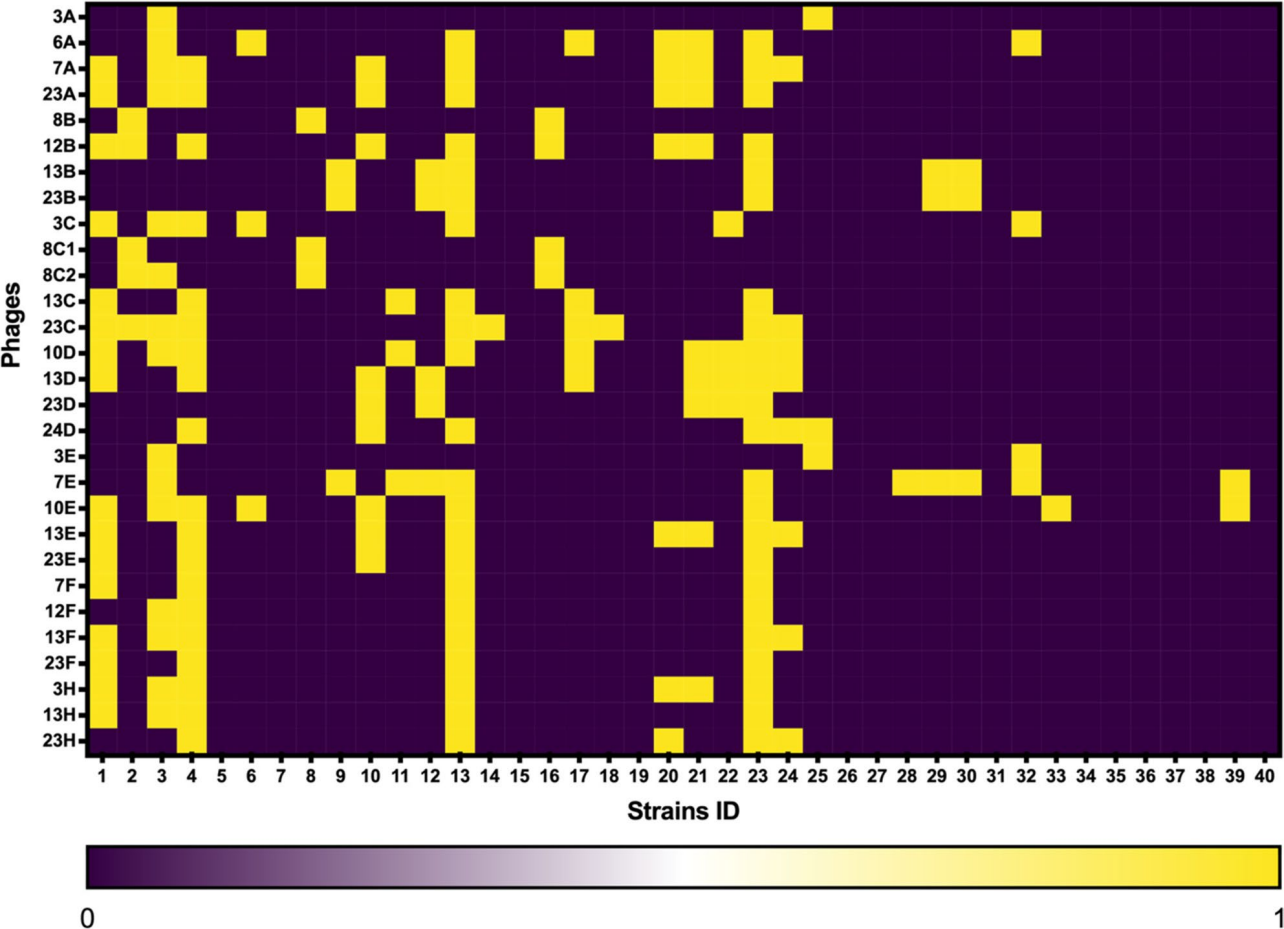
Heatmap of host range of isolated phages. The host range of each phage (rows) was determined against bacterial strains (columns) by the spot test assay. A bacterial strain was marked as susceptible and assigned a value of 1 if a clear zone of plaque formation was observed after overnight incubation. The absence of a clear zone was marked as resistant and assigned a value of 0

Given the alarming multidrug resistance patterns observed across all clinical isolates, attention was directed toward isolating and characterizing bacteriophages with lytic activity against these resistant strains as potential therapeutic agents. Sixty-nine phages were isolated from urban sewage sample. Primary screening identified 29 phage candidates capable of forming large clear plaques (Fig. 2). These candidates were purified for further characterization, and their host range was tested against 40 *A. baumannii* isolates to reveal distinct infection patterns. Phage E7 was selected for its relatively broad host spectrum, in particular, was able to infect 11 out of 40 tested strains (27.5% susceptibility) (Fig. 2).

The lytic potential of Phage E7 was further evaluated in terms of relative efficiency of plating (EOP). EOP provides a quantitative measure of phage infection efficiency across different hosts, distinguishing between high-efficiency infections (EOP ≥ 0.5) that produce robust phage yields and low-efficiency infections that may not support therapeutic amplification. Since bacterial isolate AB7 generated the highest phage titer, it was used as the indicator host for calculating the relative EOP. Except for two isolates (AB3 and AB11), where the phage produced medium EOP (< 0.5 - > 0.1) and AB33 where the phage produced low EOP (< 0.1 - > 0.01), all remaining susceptible isolates showed high EOP (> 0.5).

### Phenotypic characterization and lytic kinetics of phage E7

**Fig. 3:**
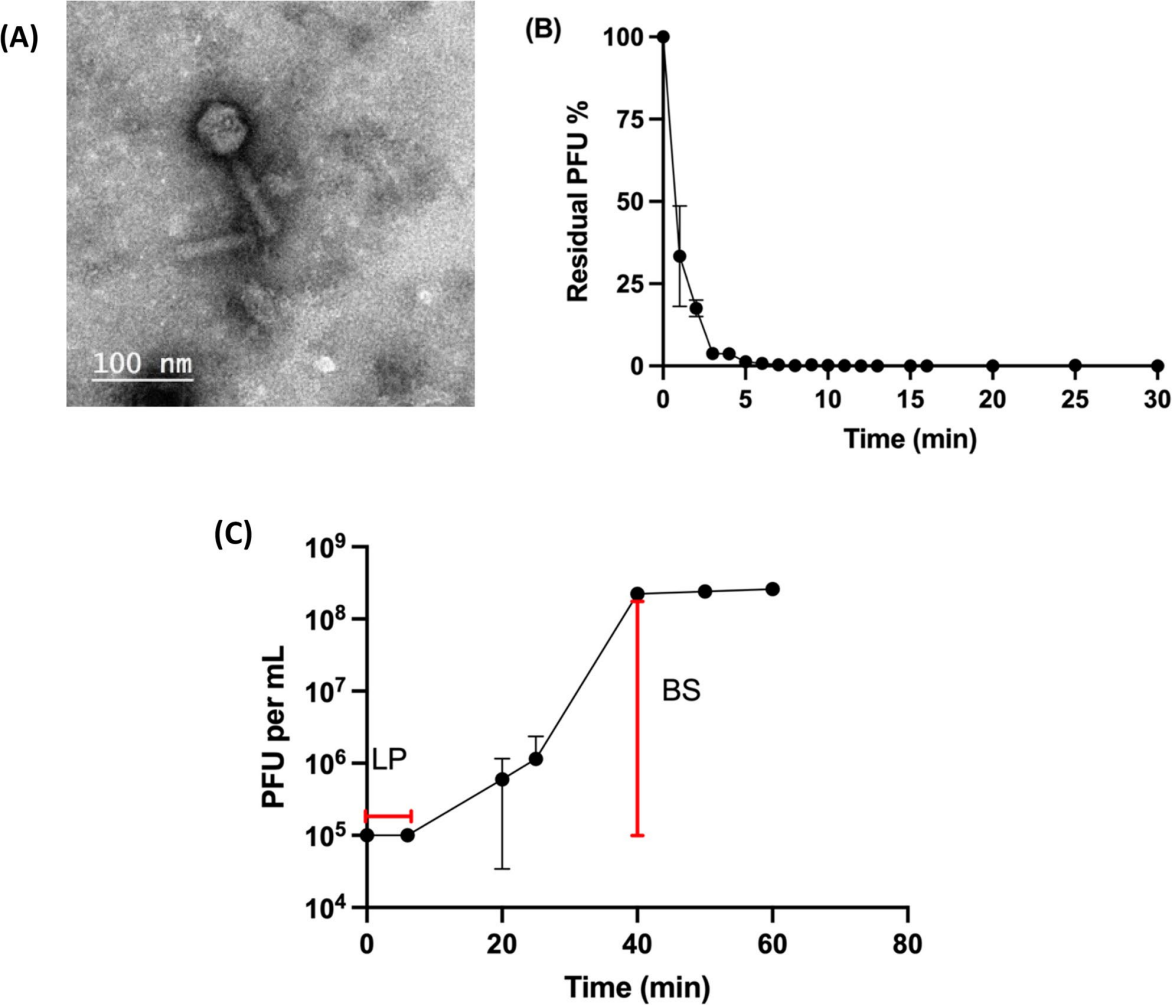
Phenotypic characterization of Phage E7. **A** Transmission electron micrograph for Phage E7.** B **Adsorption assays for Phage E7: means of triplicate assays were plotted over 30 min, with error bars indicating the standard deviation. Phage E7 adsorption reached 99% at 5 min and 100% at 7 min. **C **One-step growth curve of Phage E7 at MOI 0.001. The data represent the means of triplicates, with error bars indicating the standard deviation. The latent period lasts approximately 5 min, followed by a lysis time of around 35 min, during which bacterial cells begin to rupture until they attain a maximum, resulting in a plateau of released PFU

To comprehensively characterize the most promising candidate, we selected Phage E7 for detailed morphological, biological, and kinetic analyses based on its favorable host range and infection efficiency. Phage E7 manifested on the double-layer agar plate as transparent plaques encircled by halos of translucent regions. The phage virion was examined by TEM (Fig. 3A), displaying an icosahedral capsid of a diameter ~ 55 nm and a contractile tail of 90 nm, with terminal fibers consistent with myoviral morphology. Phage E7 demonstrated in vitro lytic activity with significant bacterial reduction of treated cultures. Host cell adsorption demonstrated that approximately 99% of the phages were adsorbed after 5 min and 100% after 7 min (Fig. 3B). A one-step growth curve indicated a latent duration of 5 min, a rise period of 35 min, and a burst size of around 1000 PFU/cell (Fig. 3C).

### *In vitro* bacteriolytic activity

**Fig. 4:**
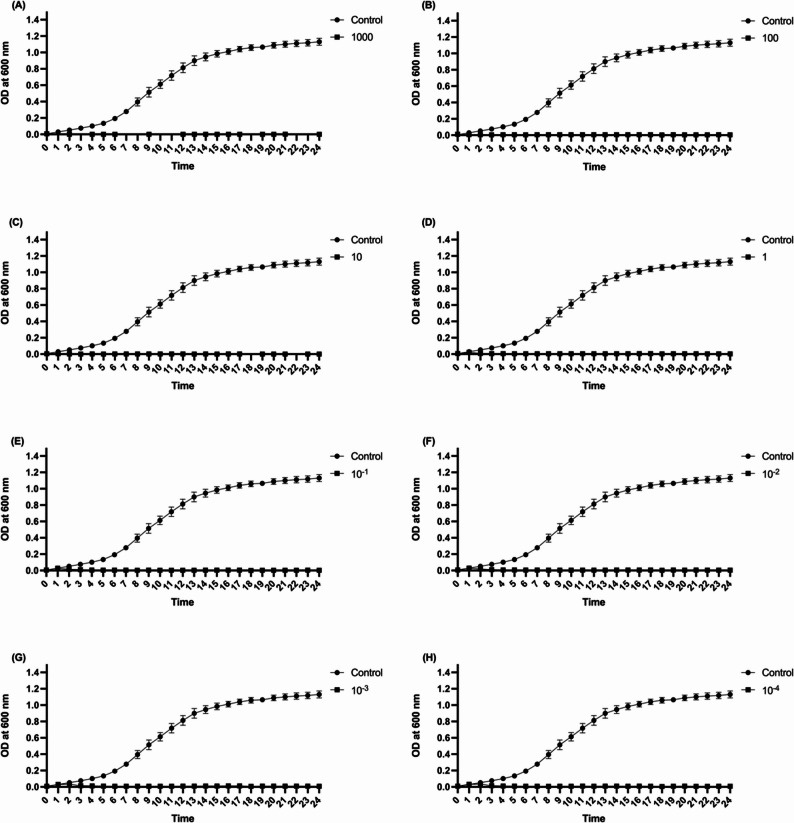
Long-term growth inhibition of isolate AB7 treated with Phage E7 at different MOIs. The following MOIs were tested: 103 (**A**), 102 (**B**), 10 (**C**), 1 (**D**), 10− 1 (**E**), 10− 2 (**F**), 10− 3 (**G**) and 10− 4 (**H**) for 24 h incubation at 37 °C. The data represent the means of triplicates; error bars indicate the standard deviation

Building upon the kinetic parameters established for Phage E7, we next evaluated its bacteriolytic efficacy across a range of multiplicities of infection to determine optimal dosing parameters for potential therapeutic applications. Phage E7 demonstrated consistent antibacterial efficacy against its host bacterium at various MOIs for 24 h. All evaluated MOIs demonstrated a significant decrease in bacterial count relative to untreated controls over the 24-hour monitoring period (Fig. 4).

### Stability of phage E7 under pH and thermal stress

**Fig. 5:**
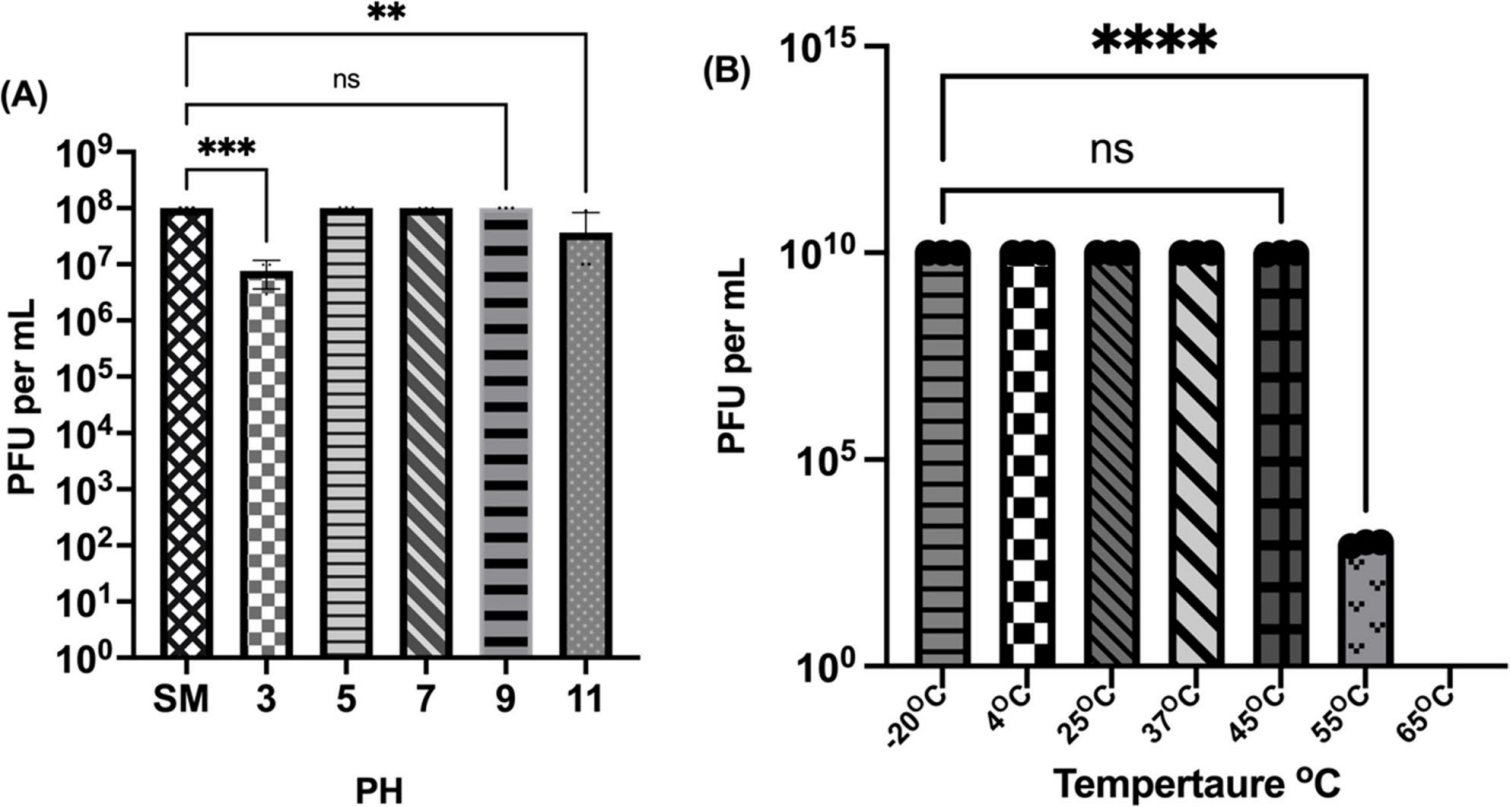
Stability of Phage E7 under different conditions. **A** Stability at different pH values after 1 h of incubation. The data represent the means of triplicates, with error bars indicating the standard deviation. GraphPad Prism (v10) was used for statistical analysis, using a one-way ANOVA test and Dunnett’s multiple comparisons test. Statistical significance is indicated by ** p ≤ 0.006 and *** p ≤ 0.0003. **B** Stability at different temperatures after 2 h of incubation. The data represent the means of triplicates, with error bars indicating the standard deviation. GraphPad Prism (v10) (GraphPad Software Inc., San Diego, CA, USA) was used to do statistical analysis, using a one-way ANOVA test and Dunnett’s multiple comparisons test. Statistical significance is indicated by **** p ≤ 0.0001. No phages survived at 65 °C

Assessing phage stability is essential for therapeutic applications, as phages may encounter adverse circumstances during storage, transit, and downstream processing. The stability of Phage E7 was assessed across a pH range of 3 to 11 after an hour and 24 h of incubation. Phage titer at pH 7 was used as control to assess the stability at various pH values. The results showed that the phage exhibited consistent lytic activity between pH 5 and pH 9 after 1 h. At pH 3 and 11, the phage’s lytic activity significantly decreased (*p* < 0.02) (Fig. 5A). The phage titers recorded after 24 h of incubation were not significantly different from those recorded after 1 h across the stable pH range of 5 to 9 (*p* > 0.05), indicating that phage E7 maintains consistent stability over extended incubation periods. For clarity and to avoid figure redundancy, only the 1-hour data are presented graphically.

The phage demonstrated significant stability across a broad temperature range from − 20 °C to 45 °C, while its activity started to decline at 55 °C and was entirely lost at 65 °C (Fig. 5B).

### Antibiofilm activity of phage E7

Considering the clinical significance of biofilm development, which enhances bacterial resistance and virulence, the antibiofilm efficacy of Phage E7 was empirically evaluated across a broad spectrum of (MOIs). The effectiveness of Phage E7 in the suppression of biofilm formation as well as the disruption of an established mature biofilm were examined by crystal violet staining and cell viability assessment.

**Fig. 6:**
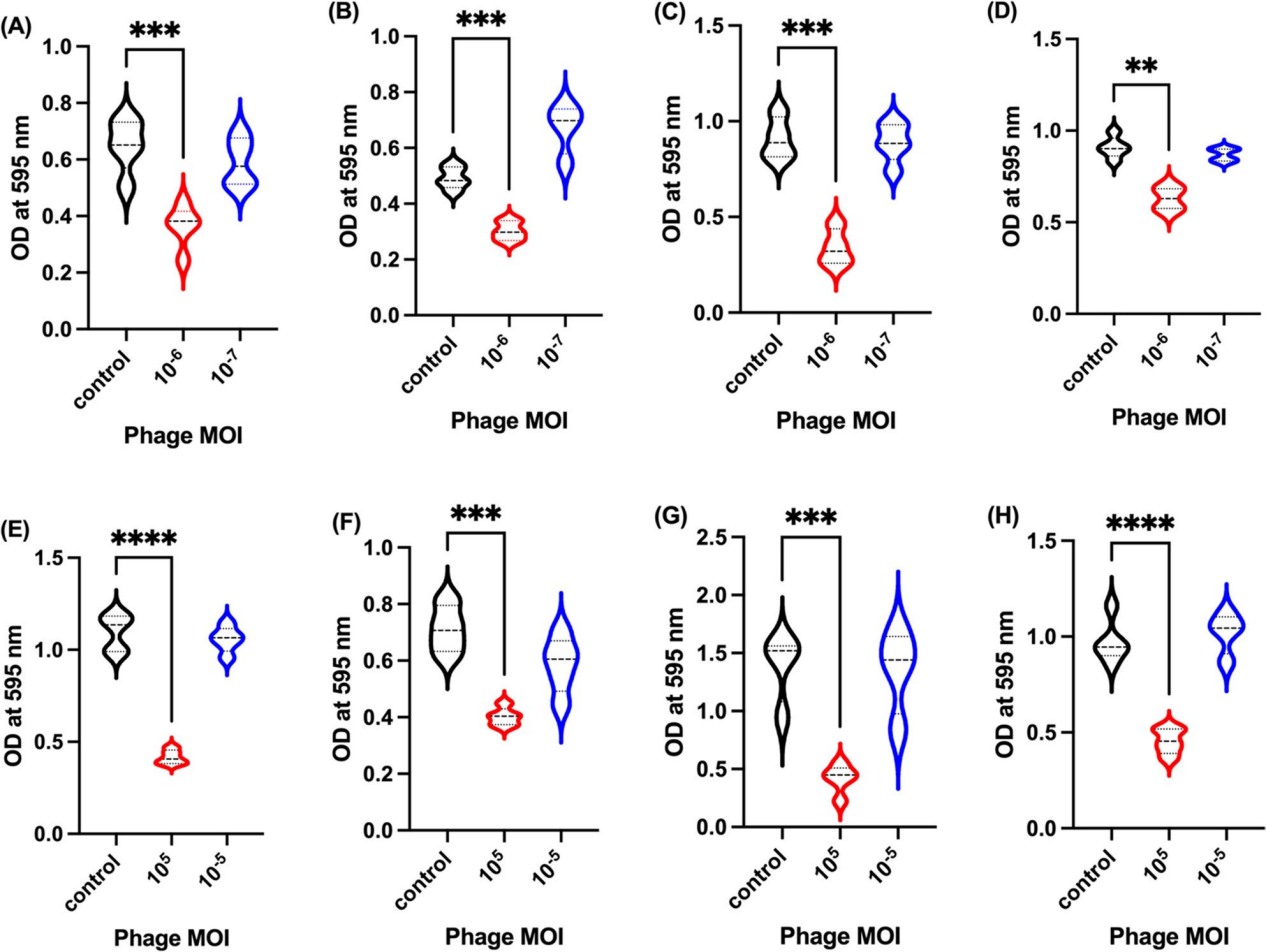
Antibiofilm activity of Phage E7 at different MOIs. **A** Biofilm inhibition for 6 h at 30 °C. **B** Biofilm inhibition for 6 h at 37 °C. (**C**) Biofilm inhibition for 24 h at 30 °C. **D** Phage E7 inhibited biofilm formation at different MOIs for 24 h at 37° (**E**) Phage E7 degraded a 48-hour pre-formed mature biofilm at different MOIs for 6 h at 30 °C. (**F**) Phage E7 degraded a 48-hour pre-formed mature biofilm at different MOIs for 6 h at 37 °C. **G** Phage E7 degraded a 48-hour pre-formed mature biofilm at different MOIs for 24 h at 30 °C. **H** Phage E7 degraded a 48-hour pre-formed mature biofilm at different MOIs for 24 h at 37 °C. GraphPad Prism (v10) (GraphPad Software Inc., San Diego, CA, USA) was used to do statistical analysis, using a one-way ANOVA test and Dunnett’s multiple comparisons test Statistically significant differences are marked by asterisks, where ** indicates p < 0.01, ***indicates p < 0.001 and ****indicates p < 0.0001

In biofilm prevention, at MOI 10− 6, Phage E7 significantly inhibited biofilm formation after 6 h of incubation at 30 °C (p < 0.0007) and 37 °C (p < 0.0005) compared to untreated controls. In contrast, MOI 10− 7 did not significantly reduce biofilm formation at 30 °C (p > 0.34) but exhibited significant inhibition at 37 °C (p < 0.005). After 24 h, MOI 10− 6 maintained significant suppression at both 30 °C (p < 0.0002) and 37 °C (p < 0.0004), whereas MOI 10− 7 had no significant effect at either temperature (30 °C: p > 0.8; 37 °C: p > 0.28) (Fig. 6).

For disruption of mature biofilm, Phage E7 demonstrated potent activity against 48-hour-old biofilms at high MOIs. Treatment with MOI 105 significantly degraded mature biofilms within 6 h at both 30 °C and 37 °C (p < 0.0001). Similarly, after 24 h, biofilm (ODs) remained significantly reduced at 30 °C (p < 0.0003) and 37 °C (p < 0.0001). In contrast, MOI 10− 5 showed no substantial disruption after 6 h (30 °C: p > 0.48; 37 °C: p > 0.08) or 24 h (30 °C: p > 0.88; 37 °C: p > 0.55) (Fig. 6).

In conclusion, Phage E7 exhibited optimal antibiofilm activity at MOI 10^− 6^ for preventing biofilm formation and MOI 10^5^ for eradicating mature biofilms, highlighting its potential as a therapeutic agent against biofilm-associated *A. baumannii* infections. 

### Genomic characterization and phylogenetic analysis of phage E7

#### Genomic analysis and annotation

To ensure the safety and therapeutic suitability of Phage E7 and to gain comprehensive insights into its genetic composition, lifestyle, and evolutionary relationships, we sequenced the genome of Phage E7 and thoroughly analyzed it. The Illumina NovaSeq platform was used to sequence the phage’s genome. Duplicates were eliminated after merging and trimming the resultant reads, then de novo assembly and annotation were performed. Phage E7 genomic sequences were added to the NCBI database under accession number PV928011.

**Fig. 7:**
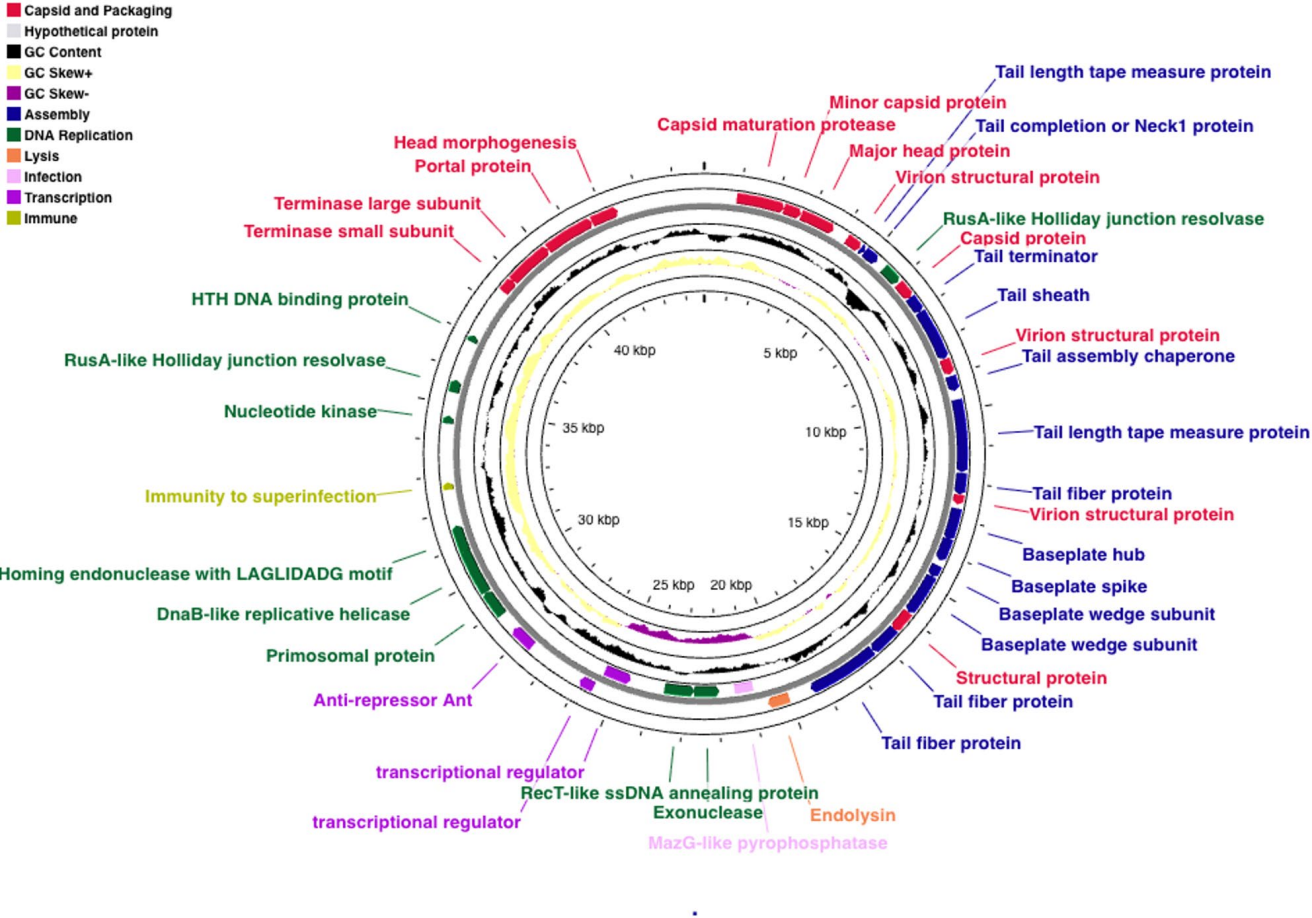
Circular genomic map of Phage E7. The color coding indicates the coding sequences (CDS), grouped by the predicted functional categories: capsid and packaging (red), assembly (blue), infection (pink), lysis (orange), DNA replication (green), immune (olive), hypothetical proteins (grey), GC content (black), and GC skew (yellow and purple). The genomics maps were created on Proksee server

The genome size of Phage E7 is 44,821 bp with an average read coverage of 101.7x and GC content of 37.8%. The genome has high coding density (95.47%), with all predicted ORFs (*n* = 83–93) initiated with an ATG start codon, except for five genes that started with GTG, one of which encoded a putative nuclease, and two that started with TTG. The largest gene is predicted to encode a tail-fiber protein (2,030 amino acids), while the smallest gene coded for a hypothetical protein of just 89 amino acids. As common among phages, the transcriptional orientation of all functionally annotated genes was located on a single strand, with the exception of a putative transcriptional regulator gene and 10 genes encoding hypothetical proteins, which were transcribed in the opposite direction (Fig. 7). Assembly quality was confirmed by DFAST, which produced a single high-quality contig with no gaps, an N50 equal to the full genome length, zero gap ratio, and an average protein length of 163.9 amino acids, characteristics collectively consistent with a compact and efficiently organized phage genome.

**Fig. 8:**
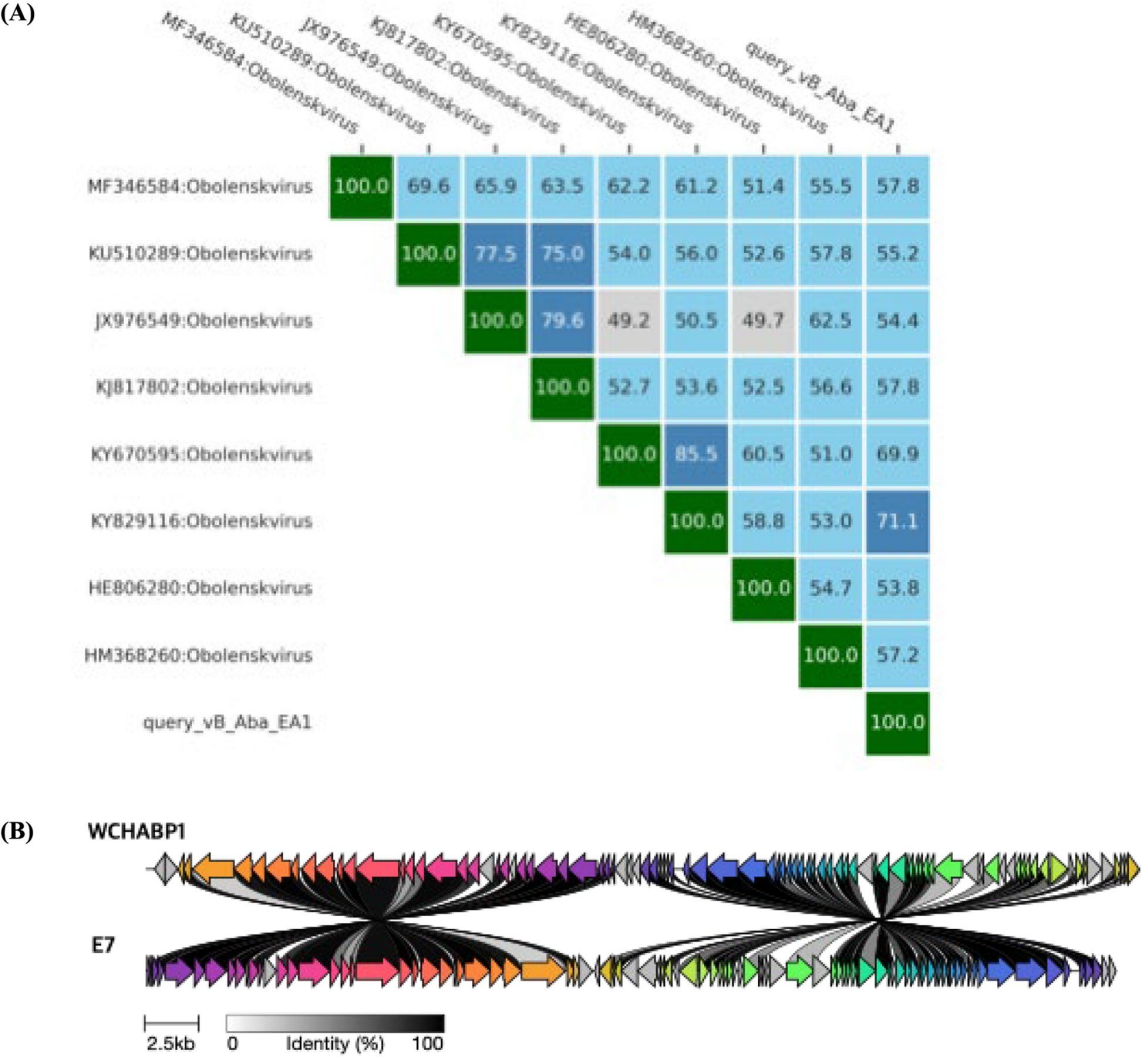
Genomic and taxonomic analysis of Phage E7. **A **Pairwise average nucleotide identity (ANI) matrix between Phage E7 and members of the Obolenskvirus genus. Phage E7 is most identical (71.1%) with Acinetobacter phage Obolenskvirus (KY829116), confirming its classification within this genus. The query phage is designated EA1 in this figure, reflecting the provisional NCBI GenBank accession record (PV928011, deposited as vB_Aba_EA1); this corresponds to phage E7 as referred to throughout this manuscript. **B** A Clinker gene cluster comparison. A comparison of whole genomes for Phage E7 and WCHABP1. Homologous coding sequences are in the same color, and the homologous coding sequences between Phage E7 and WCHABP1are linked through gray bars with the percentage amino acid identity. The identity is represented by gray bars, with darker shades of gray indicating higher percentage identity

Phage E7 was classified as a novel member of the genus *Obolenskvirus*, within the class Caudoviricetes (Fig. 8A) [32]. Comparative genomic analysis revealed its closest relative is *Acinetobacter* phage WCHABP1, with which it shares 96.2% sequence similarity and a low genetic distance (0.038). Aligning the genome of Phage E7 with *Acinetobacter* phage WCHABP1 using Clinker [64] revealed a high degree of synteny, with extensive conservation of genes involved in DNA metabolism, capsid assembly, and packaging. However, several regions of significant divergence were identified, notably between positions 15,000–20,000 bp and 30,000–35,000 bp on the Phage E7 genome.

These regions exhibit low sequence similarity of less than 30% to the reference phage (Fig. 8B). However, a broader analysis using average nucleotide identity (ANI) confirmed its highest relatedness (71.1% ANI) to the type phage of the genus, *Acinetobacter* phage Obolenskvirus (NCBI accession number KY829116), confirming its taxonomic placement (Fig. 8A).

Of the 93 proteins that the phage encodes, 25 were linked to structural and packaging functions, eight to DNA metabolism, three to transcription regulation, one to lysis, and 53 were annotated as hypothetical proteins with no currently assignable function (Fig. 7). A single putative superinfection immunity protein gene was identified at genomic position 32,630, representing the sole moron element detected in the genome. The minimal complement of moron and auxiliary metabolic genes, combined with the predominance of structural and replication-associated coding sequences, is consistent with an obligately lytic lifestyle and supports the therapeutic candidacy of phage E7, pending experimental validation. Critical to its therapeutic potential, a multi-tool screening approach was employed to maximize annotation confidence, since convergent results across tools using independent algorithms provide stronger evidence than any single tool alone. Using Pharokka, Phold, DFAST, PhageLeads, ResFinder, CARD, and VRprofile2 in combination, no genes encoding for antibiotic resistance determinants, bacterial virulence factors, tRNA genes, rRNA genes, or CRISPR elements, Anti-CRISPR proteins nor Lysogeny/integration machinery (e.g., integrases, repressors) were detected across any of the databases queried.

The absence of lysogeny-associated genetic elements was strongly supported by the BACPHLIP algorithm, which predicted a virulent lifestyle for Phage E7 with high confidence (76.25% probability). The genome was dominated by genes essential for the lytic cycle (structural, packaging, replication, and lysis), with a minimal complement of regulatory elements, which was consistent with an obligately lytic lifestyle. This combined approach improved the accuracy of functional annotations and lifestyle classification, reinforcing the therapeutic potential of Phage E7 for targeted applications. Independent confirmation by PhageScope classified 14 infection-related and 9 structural assembly domain hits within the genome, with no tRNA genes or integration machinery detected, fully consistent with the obligately lytic annotation profile established by the multi-tool pipeline described above.

A Blastp analysis of the predicted gene products revealed that 40 proteins exhibited significant similarity to known phage proteins, while 45 proteins were annotated as hypothetical proteins with no assigned function. Additionally, seven predicted genes showed no significant homology to sequences in the database and one predicted protein exhibited only weak similarity (identity < 70%). The high proportion of hypothetical proteins is not unusual for novel phages with limited representation in current databases and likely reflects the genomic novelty of phage E7 within the *Obolenskvirus* genus. Notably, the Phage E7 genome lacked rRNA, tRNA, or tmRNA genes. The analysis of transmembrane regions (TMRs) using GFF3 annotation showed no predicted classic holins, indicating that the encoded proteins likely lacked membrane-associated domains which is consistent with the annotation of a phage that may utilize a pinholin-SAR endolysin system or an alternative, non-holin based lysis mechanism.

#### Phylogenetic analysis

**Fig. 9:**
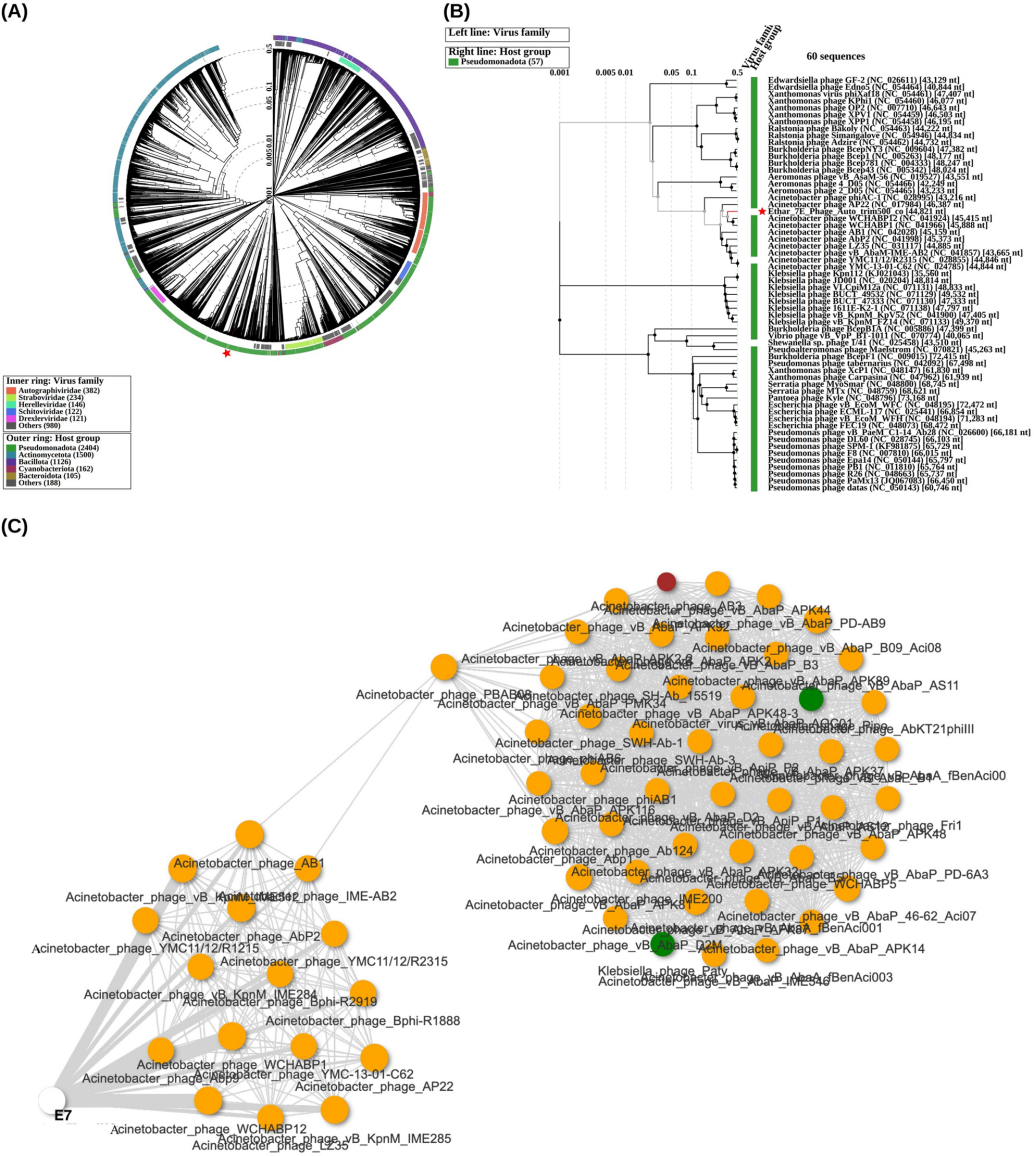
Phylogenetic analysis of Phage E7. **A **ViPtree's circular proteome tree illustrates the proteomic connections between the isolated phage (shown by red stars). and Acinetobacter phages. **B **A rectangular proteomic tree with high ViPtree similarity scores (SG > 0.5) highlighting closely related phages. **C** PhageClouds investigating the isolated phage based on intergenomic distance calculations using a threshold of 0.15 compared to phage genomes on NCBI-GenBank. The analyzed phage is highlighted with a white circle. Phages are colored according to their host species: green for Klebsiella pneumoniae, orange for Acinetobacter baumannii, and red for carbapenem-resistant A. baumannii (CRAB)

To evaluate the genomic and proteomic relations of our isolated *Acinetobacter* phage E7 to closely related phages, we conducted comprehensive phylogenetic analysis using various techniques. Using VipTree, we compared the genome of Phage E7 to 5,633 sequences of dsDNA prokaryotic viruses from a perspective of proteome-based phylogeny (Fig. 9A). To create a more focused rectangular proteomic tree, we compared the genomes of 60 phages using a proteome-based phylogenetic perspective (Fig. 9B). The genetic link between Phage E7 and other phage genomes in NCBI-GenBank was conducted using PhageCloud, an alternative protein-protein network-based method (Fig. 9C).

## Discussion

The rising incidence of infections caused by multidrug-resistant (MDR) *A. baumannii*, a threatening nosocomial pathogen, poses a formidable challenge to healthcare systems worldwide. The declining efficacy of antibiotics, including carbapenems, against these pathogens necessitates the urgent development of alternative therapeutic strategies [72, 73]. Bacteriophage therapy has regained significant interest as a promising complementary approach to combat infections caused by pan-resistant bacterial strains [74]. In this study, we successfully isolated and characterized a novel lytic bacteriophage with myoviral morphology, vB_AbaM_EA1 (E7), with demonstrated efficacy against clinical MDR *A. baumannii* isolates, in both their planktonic and biofilm states. What distinguishes Phage E7 from previously reported *Acinetobacter* phages is its exceptional combination of rapid kinetics, broad therapeutic stability, potent biofilm prevention and degradation activities, and unique genomic features that position it as a strong candidate for clinical translation.

The antibiotic resistance profile of our clinical isolates confirmed their MDR status, consistent with multiple reports about *A. baumannii* [31, 75]. The resistance to carbapenems (Meropenem) and third/fourth-generation cephalosporins confirms the critical need for effective alternatives, aligning with global reports on the rise of extensively drug-resistant *A. baumannii* [1]. More alarmingly, the complete resistance of our isolate panel to six major antibiotic classes, including last-resort carbapenems, makes them a relevant testing platform that is well representative of isolates from different intensive care settings worldwide.

Following initial isolation, we narrowed the candidate set of lysates from 69 to 29 based on plaque morphology, prioritizing phages that formed large, clear plaques. This criterion is standard practice in therapeutic phage development [35], as clear plaques indicate complete lysis, which is a phenotypic marker of a strictly lytic life cycle [76]. On the other hand, plaques with large size are often correlated with a short latent period and a large burst size [77]. Phages with these characteristics are highly efficient and can achieve rapid amplification and effective killing of the bacterial population in vivo, a crucial trait for a successful therapeutic outcome [78]. Therefore, the 29 phages selected for further study represented those with the highest potential for therapeutic application based on these desirable phenotypic characteristics.

The selection of Phage E7 was based on a lytic host range that encompassed 27.5% of the clinical strains in our panel. While this host range may initially appear modest, it is important to situate this finding within the framework of therapeutic phage development. The 27.5% susceptibility rate actually represents a clinically meaningful spectrum of activity when compared to the highly strain-specific nature of most *Acinetobacter* phages, where host ranges frequently fall below 10–15% of tested isolates. This spectrum of activity, while not universal, is considered broad for an *Acinetobacter* phage and is comparable to other therapeutically explored phages such as IsfAB78 and IsfAB39 [79], yet Phage E7 demonstrates superior kinetic parameters and antibiofilm activity compared to these reference phages, suggesting its potential utility against a clinically relevant subset of infections.

More critically, this targeted specificity could represent a therapeutic advantage rather than a limitation. Unlike broad-spectrum antibiotics that indiscriminately eliminate commensal bacteria, thereby increasing susceptibility to secondary infections, the precision of Phage E7 minimizes collateral damage to beneficial microbial communities [80]. The high efficiency of plating (EOP) on most susceptible hosts indicates robust and productive infections, a prerequisite for therapeutic efficacy [78]. Furthermore, the demonstration of high EOP values (> 0.5) across 82% of susceptible strains distinguishes Phage E7 from many reported phages that show variable infection efficiency even among permissive hosts, suggesting that Phage E7 maintains consistent lytic potency across genetically diverse *A. baumannii* strains.

Morphological analysis by TEM revealed a viral particle with an icosahedral capsid and a contractile tail, consistent with myovirus morphology. According to the latest ICTV taxonomy (2022), Phage E7 was best classified within the genus *Obolenskvirus* [40]. The observed halo formation around plaques is a phenotypic hallmark of depolymerases, enzymes that degrade capsular polysaccharides and biofilms, a feature increasingly sought after in therapeutic phages [42, 81]. The presence of these characteristic halos surrounding Phage E7 plaques appears to indicate antibiofilm activity, which was later confirmed experimentally. The in vitro pharmacokinetic profile of Phage E7 reveals advantages that distinguish it from the majority of reported *Acinetobacter* phages. Its favorable in vitro pharmacokinetics, including rapid adsorption kinetics (5 min), a short latent period (5 min), and a large burst size (~ 1000 PFU/cell), suggest a highly efficient lytic cycle capable of achieving rapid bacteriolysis.

With 99% adsorption within 5 min, Phage E7 ranks among the fastest-adsorbing *Acinetobacter* phages characterized to date. These in vitro kinetics are superior to those reported for phage vB_AbaS_Loki (40 min latent period) [82] and phage *phi G7* (20 min latent period) [83], yet Phage E7’s burst size of approximately 1000 PFU/cell exceeds the typical range of 50–300 PFU/cell reported for most *Acinetobacter* phages, suggesting superior replicative efficiency that translates to more rapid bacterial clearance in therapeutic applications. The in vitro time-kill assays corroborated this, showing a rapid, massive reduction in bacterial load at all MOIs, further solidifying its potent lytic capability. Specifically, the ability of Phage E7 to maintain suppression over 24 h at MOIs as low as 10^− 4^ demonstrates exceptional bactericidal efficiency. This finding is particularly notable when compared to a systematic network meta-analysis concluded that no single antibiotic reliably produces sustained bactericidal effect against MDR *A. baumannii*, necessitating combination therapy [84]. In this context, the ability of Phage E7 to function as a single agent with sustained activity over 24 h compares favorably with the limited efficacy of conventional antibiotic regimens.

A critical consideration for any therapeutic phage is its stability under physiological and storage conditions. Phage E7’s stability profile addresses a significant obstacle in phage therapy formulation and storage stability. E7 exhibited remarkable resilience, maintaining activity across a wide range of temperatures (-20 °C to 45 °C) and pH values [5–9]. This broad stability window is consistent with findings for phages such as pIsf-AB02 [43]; however, E7’s retention of full activity at -20 °C and its stability up to 45 °C for 2 h without significant titer loss represents higher thermal tolerance than most characterized phages [43, 82], which typically show substantial activity decline above 40 °C or require specialized cryopreservation protocols.

Such stability profile is highly encouraging for pharmaceutical development, suggesting that Phage E7 could remain viable during storage, transport, and upon administration through various routes. The pH stability range of 5–9 is particularly significant as it encompasses the pH environments encountered in different infection sites, including acidic wound environments (pH 5.5–6.5), neutral bloodstream (pH 7.4), and slightly alkaline conditions that may occur in biofilm microenvironments. This physiological pH tolerance suggests that Phage E7 could maintain therapeutic efficacy across diverse anatomical sites without requiring complex formulation strategies, a practical advantage that simplifies clinical implementation and reduces manufacturing costs compared to pH-sensitive phages requiring buffered delivery systems.

A particularly clinically significant aspect of this study was the demonstration of Phage E7’s potent antibiofilm activity. Biofilms are a cornerstone of *A. baumannii*’s pathogenicity, conferring enhanced resistance to antibiotics and host immune responses [13]. Biofilm-associated *A. baumannii* infections are notoriously difficult to eradicate, as biofilm-embedded cells can tolerate antibiotic concentrations up to 1,000-fold higher than their planktonic counterparts, and biofilm formation has been directly associated with increased mortality in ICU patients [13, 85]. The development of antibiofilm strategies therefore represents a critical clinical need in the management of *A. baumannii* infections.

Unlike most reported phages that demonstrate either biofilm prevention or disruption capabilities, but rarely both effectively, Phage E7 has potent activity in both modes. While the phage cocktail of phages B3 and 404ad reported by Blasco et al. (2022) achieved both prevention and disruption of *A. baumannii* biofilms, this dual activity required a combination of two phages rather than a single isolate [45]. The demonstration of both activities within phage E7 alone therefore represents a meaningful advance over previously reported single-phage preparations targeting *A. baumannii* biofilms.

It significantly inhibited the formation of new biofilms at remarkably low MOI (10⁻⁶), achieving statistically significant suppression (*p* < 0.0005) at both 30 °C and 37 °C within 6 h. This prevention efficacy at MOI 10⁻⁶ is particularly noteworthy when compared to published data: Su et al. (2025) reported that phage vB_AbaM_AB4P2, a depolymerase-encoding *A. baumannii* myovirus, required MOIs of 0.1 to 1 to achieve comparable biofilm inhibition [42], suggesting that phage E7 achieves significant antibiofilm activity at substantially lower phage concentrations, which has direct implications for dose optimization in therapeutic applications.

More impressively, phage E7 effectively disrupted mature, 48-hour old biofilms. The choice of the 48-hour timepoint is clinically relevant: the majority of published phage antibiofilm studies against *A. baumannii* assess disruption of 24-hour biofilms [42] which represent an earlier and less architecturally complex maturation stage. *A. baumannii* biofilms at 48 h develop extensive extracellular polymeric substance (EPS) networks and a higher proportion of metabolically dormant persister cells that confer substantially greater tolerance to both antibiotics and phage-mediated lysis compared to 24-hour biofilms [86]. The ability of phage E7 to achieve significant disruption (*p* < 0.0001) of these more mature and resilient biofilm structures therefore represents a higher standard of antibiofilm efficacy than that demonstrated by many previously reported *A. baumannii* phages, including phage vB_AbaS_Loki and phage IME200, which showed limited activity against biofilms older than 24 h [82].

The requirement for a high MOI (10^5^) to eradicate established biofilms is consistent with the biophysical challenges inherent to biofilm architecture and does not diminish Phage E7’s therapeutic value; rather, it reflects the realistic dosing requirements for treating established infections, largely attributed to constrained phage diffusion and the metabolic heterogeneity of cells within the biofilm matrix, which can confer a transient tolerance to phage-mediated lysis [86]. This MOI requirement is consistent with findings reported for other antibiofilm phages targeting *A. baumannii*: Blasco et al., similarly required high phage concentrations to achieve disruption of mature *A. baumannii* biofilms even when using a two-phage cocktail [45], and Su et al., reported that high MOIs were necessary for effective disruption of established *A. baumannii* biofilms by phage vB_AbaM_AB4P2 [42]. The consistent requirement for higher MOIs in biofilm disruption compared to biofilm prevention across multiple studies reflects the fundamental biophysical barrier posed by the mature biofilm matrix rather than a limitation specific to phage E7.

Importantly, the observation that Phage E7 maintained antibiofilm efficacy at both 30 °C and 37 °C indicates functional versatility across temperature ranges encountered in superficial wounds vs. deep-tissue infections, a feature rarely systematically evaluated but critically important for treating the diverse anatomical sites colonized by *A. baumannii*.

The putative depolymerase activity of Phage E7 likely facilitates biofilm penetration by degrading the extracellular polymeric substance, a major constituent of the biofilm architecture [81]. Genomic analysis identified a large tail fiber-associated protein (2,030 amino acids) containing putative depolymerase domains, providing a molecular mechanism for the observed antibiofilm activity. This unusually large tail protein may confer enhanced biofilm-degrading capacity through multiple catalytic domains or extended reach into the biofilm matrix, warranting detailed biochemical characterization in future studies. This dual antibiofilm activity is of significant clinical importance, as it demonstrates potential for both preventing infections on medical implants and treating established biofilm-associated chronic wounds. While the ability to either prevent biofilm formation or disrupt mature biofilms has been documented for other *Acinetobacter* phages, such as phage B3, phage 404ad, and the cocktail of both phages [45], the demonstration of both activities within a single phage is a notable strength of Phage E7.

Furthermore, the efficacy of Phage E7 against 48-hour old mature biofilms at a high MOI is a particularly robust finding. Many studies assess biofilm disruption at earlier time points (e.g., 24 h); the effectiveness against a more established, and thus more resilient, biofilm structure suggests that Phage E7, potentially aided by its putative depolymerase activity, possesses a potent biofilm-degrading capability. This comprehensive antibiofilm profile makes Phage E7 well suited for treating ventilator-associated pneumonia, catheter-related bloodstream infections, and chronic wound infections (the three most clinically challenging biofilm-associated *A. baumannii* infection syndromes).

The genomic characterization of Phage E7 revealed unique molecular features that not only ensure its safety profile, but also provide mechanistic insights into its superior phenotypic characteristics. Classified as a novel member of the *Obolenskvirus* genus, E7 has genomics features that distinguish it from its closest relatives. While it is highly similar to *Acinetobacter* phage WCHABP1 at the DNA level, the presence of unique genes in Phage E7 specifically, those encoding a distinct putative tail fiber protein, an additional contractile tail fiber protein (Gp17), and a putative superinfection immunity protein. The fact that these genes are not found in WCHABP1 defines the novelty of Phage E7 and provides it with distinct functional advantages. These genetic distinctions provide a plausible explanation for its phenotypic characteristics: the unique tail fiber proteins may be responsible for its specific host range and efficiency [87], and the superior adsorption kinetics compared to other *Obolenskvirus* members, potentially through enhanced receptor-binding affinity or multi-receptor recognition capability. The presence of a superinfection immunity protein serves a different purpose. This protein prevents a bacterial cell that is already infected from being reinfected by a second, related phage. This mechanism ensures that the host cell’s resources are used entirely to produce progeny of the first phage rather than being wasted on competing infections [88]. In Phage E7, this may help explain its unusually high burst size of approximately 1,000 PFU per cell, which is notably larger than what is typical for many *Acinetobacter* phages. Furthermore, a broader analysis using average nucleotide identity (ANI) confirmed that while related, Phage E7 forms a distinct branch within the genus, sharing only 71.1% ANI with the type phage, *Acinetobacter* phage Obolenskvirus. This genetic distance confirms its status as a novel viral isolate.

Most importantly, comparative genomics reinforced the absence of any genes associated with bacterial virulence factors, antibiotic resistance determinants, tRNA genes, or integrases. The absence of these undesirable genetic elements, combined with the high-confidence prediction of a strictly lytic lifestyle by the BACPHLIP algorithm, strongly supports its safety profile and mitigating potential risks associated with horizontal gene transfer. The genome is predominantly dedicated to structural, packaging, replication, and lysis functions, hallmarks of an obligately lytic phage suited for therapeutic applications.

In conclusion, through a structured pipeline of phenotypic and genotypic analyses, we characterized Phage E7 as a therapeutically superior candidate that addresses multiple limitations observed in previously reported *Acinetobacter* phages. The convergence of exceptional kinetic parameters (5-minute adsorption, 5-minute latent period, ~ 1000 PFU/cell burst size), dual-mode antibiofilm activity (effective at both preventing formation and disrupting 48-hour mature biofilms), broad physicochemical stability (pH 5–9, -20 °C to 45 °C), clinically relevant host range (27.5% of MDR isolates), and comprehensive genomic safety profile collectively position Phage E7 as a promising candidate for clinical translation.

Furthermore, the practical advantages of Phage E7’s stability profile significantly de-risk pharmaceutical development, while its potent biofilm-disrupting capacity directly addresses the most clinically intractable aspect of *A. baumannii* infections that contributes to treatment failure in ICU settings [85].

Future work will focus on in vivo validation of its efficacy and safety in animal models, including murine pneumonia and wound infection models that recapitulate the biofilm-associated pathology characteristic of clinical *A. baumannii* infections, alone and in combination with antibiotics, to evaluate potential synergistic interactions that could enable antibiotic resensitization or dose reduction strategies, to pave the way for its potential use in combating devastating CRAB infections.

Additionally, scaled production under Good Manufacturing Practice (GMP) conditions and comprehensive pharmacokinetic/pharmacodynamic (PK/PD) studies will be essential next steps to advance Phage E7 toward Phase I clinical trials, with the ultimate goal of providing clinicians with a validated therapeutic option for infections that currently have no effective treatment alternatives.

## Data Availability

The datasets generated and analyzed during the current study are available from the corresponding author upon request. The genome sequence of the bacteriophage described in this study has been deposited in NCBI database under accession number PV928011. The bacterial strains used are available from the corresponding author upon reasonable request.
